# Cyberbiosecurity in high-containment laboratories

**DOI:** 10.3389/fbioe.2023.1240281

**Published:** 2023-07-25

**Authors:** Elizabeth Crawford, Adam Bobrow, Landy Sun, Sridevi Joshi, Viji Vijayan, Stuart Blacksell, Gautham Venugopalan, Nicole Tensmeyer

**Affiliations:** ^1^ Gryphon Scientific, Takoma Park, MD, United States; ^2^ Veribo Analytics, Bethesda, MD, United States; ^3^ Praxis Biorisk Systems, Singapore, Singapore; ^4^ Mahidol-Oxford Tropical Research Medicine Unit, Faculty of Tropical Medicine, Mahidol University, Bangkok, Thailand; ^5^ Centre for Tropical Medicine and Global Health, Nuffield Department of Medicine, Nuffield Department of Medicine Research Building, University of Oxford, Oxford, United Kingdom

**Keywords:** cyberbiosecurity, cybersecurity, biosecurity, biosafety, risk assessment, high-containment laboratories (HCLs)

## Abstract

High-containment laboratories (HCLs) conduct critical research on infectious diseases, provide diagnostic services, and produce vaccines for the world’s most dangerous pathogens, often called high-consequence pathogens (HCPs). The modernization of HCLs has led to an increasingly cyber-connected laboratory infrastructure. The unique cyberphysical elements of these laboratories and the critical data they generate pose cybersecurity concerns specific to these laboratories. Cyberbiosecurity, the discipline devoted to the study of cybersecurity risks in conjunction with biological risks, is a relatively new field for which few approaches have been developed to identify, assess, and mitigate cyber risks in biological research and diagnostic environments. This study provides a novel approach for cybersecurity risk assessment and identification of risk mitigation measures by applying an asset-impact analysis to the unique environment of HCLs. First, we identified the common cyber and cyberphysical systems in HCLs, summarizing the typical cyber-workflow. We then analyzed the potential adverse outcomes arising from a compromise of these cyber and cyberphysical systems, broadly categorizing potential consequences as relevant to scientific advancement, public health, worker safety, security, and the financial wellbeing of these laboratories. Finally, we discussed potential risk mitigation strategies, leaning heavily on the cybersecurity materials produced by the Center for Internet Security (CIS), including the CIS Controls^®^, that can serve as a guide for HCL operators to begin the process of implementing risk mitigation measures to reduce their cyberbiorisk and considering the integration of cyber risk management into existing biorisk management practices. This paper provides a discussion to raise awareness among laboratory decision-makers of these critical risks to safety and security within HCLs. Furthermore, this paper can serve as a guide for evaluating cyberbiorisks specific to a laboratory by identifying cyber-connected assets and the impacts associated with a compromise of those assets.

## Introduction

In the life sciences, the digitalization of research and development has enabled the creation of new techniques and tools, increasing the efficiency of project design and implementation (Peters, 2012; Krüger et al., 2020). In particular, biological laboratories benefit from the automation and digitalization of laboratory infrastructure, including elements such as the instruments used for data collection and analysis or electronic laboratory notebooks and data storage (Perkel, 2017). For example, in diagnostic laboratories and healthcare institutions, increased automation of laboratory instruments has expedited the diagnostic process, increasing the throughput capabilities of these facilities, and providing patients with their test results faster (Lippi and Da Rin, 2019). The potential for new innovation resulting from integrating technological advancements in biological laboratories could significantly improve people’s health and lives. However, with the increased digitalization and technological advances in the biological sciences comes the emergence of new security risks and their related consequences. In the context of laboratories, the increased cyber-connectedness of biological laboratories has resulted in an increased risk from cyber attacks, and the emergence of additional potential consequences resulting from such attacks. This issue remains underappreciated and poorly addressed in the scientific community.

Cyber attacks have increased in frequency over the last few years, with most organizations worldwide experiencing regular attacks, severely affecting the global economy (AAG Digital, 2019). These attacks have resulted in a greater focus on cybersecurity, defined in the National Institute of Standards and Technology (NIST) Cybersecurity Framework as the “process of protecting information by preventing, detecting, and responding to (cyber) attacks.” The growing number of cyber attacks on institutions in the life sciences has increased awareness and led to the emergence of a new area of study termed cyberbiosecurity (Check Point Research, 2022). Cyberbiosecurity is the process of identifying and assessing the risks within or at the interfaces of cybersecurity, cyberphysical security, biosecurity, and biosafety and developing and implementing mitigation measures to prevent, detect, respond, and recover from incidents (Murch et al., 2018). Understanding the implications of cyberbiosecurity requires an understanding of the relevant disciplines from which it converges: cybersecurity and biorisk management. Biorisk management comprises two related but distinct concepts, biosecurity and biosafety. Biosecurity is an evolving concept in the life sciences community; this paper defines biosecurity as the measures used to prevent the “unauthorized access, loss, theft, misuse, diversion, or release” of biological or related materials (WHO, 2020a). Biosafety relates to the measures used to prevent the “unintentional exposure to biological agents or their inadvertent release.” (WHO, 2020a). Evaluating and subsequently addressing cyber risks in biological laboratories requires understanding the risks considered in each discipline, such as safety, security, and public health.

Biological laboratories that work with dangerous pathogens have increased biosafety and biosecurity risks compared to other laboratories. While there are unique nuances concerning the classification of pathogens utilized at the individual laboratory level, generally, pathogens are defined by Risk Group, where pathogens belonging to Risk Groups 3 and 4 are often called high-consequence pathogens (HCPs) and require the most extensive containment precautions (WHO, 2020a). These groups include pathogens that cause severe or lethal diseases such as Ebola, tuberculosis, or plague. Laboratories working with HCPs are usually designated as Biosafety level (BSL)-3 or BSL-4 and are collectively referred to as high-containment laboratories (HCLs) (Yeh et al., 2021). These laboratories perform critical and timely research on infectious diseases, provide diagnostic services, and produce vaccines for HCPs; these services are essential to society, and many HCLs are considered critical infrastructure (Reed and Dunaway, 2019). Because HCLs house HCPs and their associated data and may function as part of critical infrastructure, these laboratories must have enhanced safety and security measures under the norms promulgated by international standards (WHO, 2020b). However, the increased safety and security measures currently outlined in most open source biorisk management guidance do not extend to include cyberbiosecurity considerations associated with HCLs.

Research into the threats, risks, vulnerabilities, and consequences associated with cyberbiosecurity is relatively new, and much of the threat landscape remains to be characterized. Reed and Dunaway, (2019) introduced discourse on cyberbiosecurity in laboratories, generally addressing additional risks in BSL-2, BSL-3, and BSL-4 laboratories by identifying trends that could lead to added vulnerabilities and threats in the future (Reed and Dunaway, 2019). Here, we expound upon this foundation, providing an in-depth assessment of vulnerabilities and risks for each type of HCL and identifying both cyber and physical measures to mitigate these risks. Specifically, we 1) explore examples of historical incidents that highlight the relevance of cybersecurity to HCLs, 2) identify key assets in HCLs that contribute to their risks and vulnerabilities, an exercise foundational to performing an asset-impact analysis (see methods); 3) analyze and categorize risks and consequences that may result from a cyber incident, categorized broadly as financial, public health, worker safety, security, and scientific advancement impacts; and 4) discuss the need for cyber risk management as part of a biorisk management program.

## Methods

### Identifying historical events

We conducted a literature review of historical incidents of cyber attacks to understand the known cyber vulnerabilities and contextualize the current threat environment in the context of cyberbiosecurity in HCLs. This literature review included news sources, government reports, grey literature, and peer-reviewed literature, all of which were searched using keywords to identify any recent high-consequence cyber attack. The keywords focused on laboratories, the life sciences, and cyberphysical systems. Examples were included in this paper if they highlighted vulnerabilities relevant to the cyberbiosecurity of HCLs The results from the literature are included in Supplementary Table S1. While the examples provided demonstrate known vulnerabilities and potential consequences of successful cyber attacks in HCLs, they do not provide a comprehensive description of historical events as many cyber attacks are not disclosed in the public domain.

### Asset-impact analysis

**FIGURE 1:**
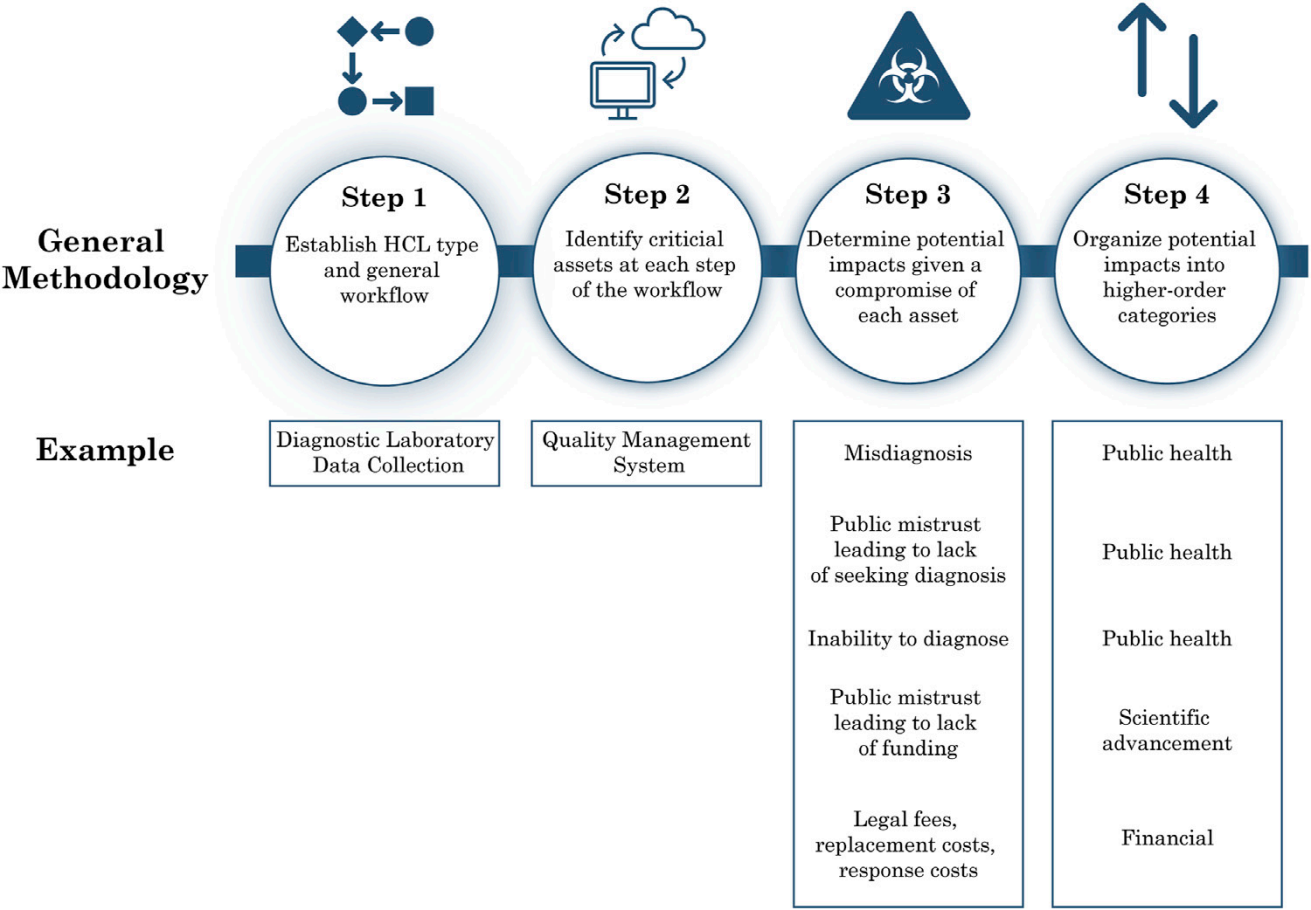
Asset-impact analysis methodology summary. Graphic showing methodology used for asset-impact analysis applied to HCLs.

To characterize risks in the context of cyberbiosecurity in HCLs, we applied a qualitative, asset-impact risk analysis approach described in the NIST Guide for Conducting Risk Assessments (Ross, 2012). An asset-impact analysis includes identifying existing cyber or cyberphysical systems, determining the value of these assets within the organization, assessing the associated vulnerabilities due to these assets, and analyzing the impacts which would stem from compromise of the assets. To tailor this analysis approach to the context of cyberbiosecurity in HCLs, we first performed a nonsystematic literature review to determine the general cyber-workflows and common cyber and cyber-physical assets of research, diagnostic, and biomanufacturing HCLs. We then systematically identified the potential adverse outcomes that could result from the compromise of each asset, considering consequences due to a loss of confidentiality, integrity, or availability, summarized as unauthorized access, unauthorized alteration, or prevention of the use of the asset, respectively. To evaluate potential impacts due to compromise of each asset, we: 1) determined the cyber-connectivity that is possible for each asset type (e.g., we considered the storage systems with advanced options for connectivity including temperature monitoring and sample inventory rather than a basic freezer); 2) estimated the value provided by each asset that could be lost due to a cyber incident, including value lost to the organization, scientific advancement, and the public; 3) determined potential down-stream consequences from cyber-incidents that could occur due to the nature of the work done in an HCL (e.g., we considered biosafety and biosecurity risks of HCPs and incorporated those risks into our evaluations). The resulting dataset of workflows, assets, and adverse outcomes was further evaluated to identify larger areas of impact associated with cyber incidents in HCLs. The steps included in the asset-impact analysis are summarized in Figure 1. References used for determining the workflow and performing the asset-impact analysis are found in Supplementary Table S1.

### Cyberbiorisk management

We performed a literature review to identify common risk management practices for cybersecurity, biosecurity, and biosafety, as well as existing literature on cyberbiosecurity. To inform our discussion, we analyzed similarities and differences in risk management practices within these fields. References which identify relevant risk management practices are found in Supplementary Table S1.

## Known cyber vulnerabilities and previous cyber incidents in laboratories

Cyber attacks have been increasing in frequency and sophistication in recent years (Check Point Research, 2022). In a cybersecurity survey conducted by McAfee, only 4% of 1,500 companies reported that they did not experience a cyber incident in 2019 (Smith and Lostri, 2021). According to Check Point Research, the “Education/Research” sector was the most targeted, with an average of 1,605 weekly attacks per organization in 2021, increasing 75% from 2020 (Check Point Research, 2022). The consequences of cybercrimes take many forms and can have impacts reaching beyond the organization directly affected. Examples include but are not limited to opportunity costs, remediation costs, losses from productivity, system downtime, data loss, shortages of critical medical supplies, and loss of public trust. The total economic cost of global cybercrime was estimated to be over $1 trillion dollars as of 2020, according to estimates by McAfee (Smith and Lostri, 2021).

Historical incidents can provide real-world examples of the consequences of cyber attacks, including those targeted at specific organizations or untargeted and sent out indiscriminately to many organizations (Biju et al., 2019). We note that while targeted attacks are less common than untargeted attacks, certain industries, including education, research, manufacturing, and healthcare, among others, experience targeted attacks more frequently than others (Kessem, 2021). Some recent examples are included in the following discussion.

Biological laboratories, including HCLs, perform critical diagnostic functions and producing essential vaccines and therapeutics. Cyber attacks compromising essential laboratory and biomanufacturing functions can have significant consequences, such as shortages of essential drugs and vaccines. For example, the pharmaceutical company Merck was hit by the NotPetya attack in 2017 (MDL, 2017). This attack temporarily shut down several essential operations throughout the company for several months, including the production of several drugs and vaccines (Henriquez, 2022). In this case, the United States Center for Disease Control (CDC) stockpiles and other manufacturers were able to meet the consumer demand for HPV and Hepatitis vaccines despite the loss of production capacity (Henriquez, 2022). However, the incident illustrates how future cyber attacks could result in shortages of essential vaccines and therapeutics. Downtime of critical research or diagnostic laboratories could be similarly disruptive, particularly in laboratories with unique capabilities for their geographic region.

Many HCLs produce data relevant to public health, such as data that informs the manufacture of essential vaccines and therapeutics. Maintaining the confidentiality and integrity of these data is critical for the data to be trusted by regulators and the public. Laboratories are also often ethically and legally required to maintain confidentiality of critical data. Cyber attacks that compromise critical data could undermine public trust in the institution or its products. In 2021, the European Medicines Agency (EMA), a regulatory agency responsible for overseeing and approving the development of COVID-19 vaccines in Europe, suffered a targeted attack suspected to be a misinformation campaign involving COVID-19 vaccines (Cerulus, 2021). Data stored on an EMA server included email screenshots, EMA peer review comments, technical documents, and presentations relating to the regulatory submission for Pfizer and BioNTech’s COVID-19 vaccine candidate BNT162b2 (Cerulus, 2021). These data were accessed, manipulated, and leaked by hackers (Cerulus, 2021). Future leaks of manipulated data could similarly result in a loss of public trust in vaccines.

HCLs may also use and produce data of strategic financial value, including intellectual property (IP) or trade secrets. Cyber attacks resulting in unauthorized access to this information could result in significant financial impacts. A cyber attack campaign known as Epic Turla or Uroboros was discovered in 2014 (Global Research and Analysis Team, Kaspersky Lab, 2014). Among the targeted institutions were research and pharmaceutical production facilities located primarily in Europe and the Middle East (Global Research and Analysis Team, Kaspersky Lab, 2014). This attack successfully stole IP from pharmaceutical and research organizations, demonstrating the risks to IP and other important research data posed by cyber incidents (Global Research and Analysis Team, Kaspersky Lab, 2014).

HCLs also rely on cyberphysical systems (CPSs) for a variety of functions. CPSs integrate cyber-based control mechanisms into physical infrastructure; CPSs in many industries often pose a significant risk due to cyber attacks. In HCLs, examples of CPSs include the building automation system (BAS) and certain types of data collection and analysis instruments. A cyber attack resulting in the compromise of CPSs within HCLs could lead to a multitude of adverse outcomes, including laboratory downtime, breach of containment, or diagnostic errors, depending on the context. In 2021, hackers targeted the University of Oxford’s Division of Structural Biology research laboratory, gained access to several CPSs, and demonstrated the ability to control pumps and pressure, including disabling a pressure alarm (Brewster, 2021; Osborne, 2021). Although this incident did not occur in an HCL, it demonstrates the ability of malicious actors to tamper with cyber-connected laboratory equipment and cyberphysical systems remotely.

These real-world examples demonstrate known vulnerabilities and their associated negative impacts and can provide insights into the potential risks that HCLs may encounter. The realization of such risks in these examples supports the importance of assessing the entire spectrum of cyber risks in HCLs and proactively applying appropriate risk mitigation strategies to reduce both the likelihood and severity of a cyber attack.

## Cyber considerations in HCLs

These historical incidents highlight many potential impacts of cyber attacks on HCLs. Understanding potential cyber risks in HCLs requires a foundational understanding of the existing cyber and cyberphysical systems contained within the lab. Working with HCPs requires the implementation of enhanced containment precautions and additional security measures, measures which are often controlled by or connected to CPSs within the laboratory (Gao et al., 2021). Although the cyber-workflow of each individual laboratory is distinct, some general types exist with similar workflows and purposes. Most HCLs worldwide, including government, academic, and private institutions, fit within one of three groups: research laboratories, diagnostic laboratories, and biomanufacturing facilities. In this paper, we focus our initial work on analyzing workflows and risks in laboratories studying human pathogens without the use of experimental animal work. Although many of these findings might be generalizable to animal facilities (ABSL and BSL Ag facilities) and to those handling pathogens with agricultural impact, this paper only assesses the cyber biorisks associated with HCLs working with human pathogens and that do not work with live animals. Additional work would be required to account for these unique workflows and potential cyber risks.

The section below describes common cyber and cyberphysical systems found in HCLs and discusses their use within the laboratory. We first focus on commonalities between the three overarching types of HCLs, then briefly describe the unique considerations of research, diagnostic, and biomanufacturing laboratories specifically. This section describes the typical cyber-connected assets and the points of entry or attack pathways introduced because of the connection of these assets to computer networks. The following section uses this foundational identification of assets to analyze the potential impacts of cyber incidents in HCLs.

### Cyber elements of high-containment laboratories

The specific workflow and assets of research labs are tailored to their subject matter area and experimental design but can generally be summarized into the following steps: project planning, pathogen research, data collection, data analysis, and data storage and communications.

**FIGURE 2:**
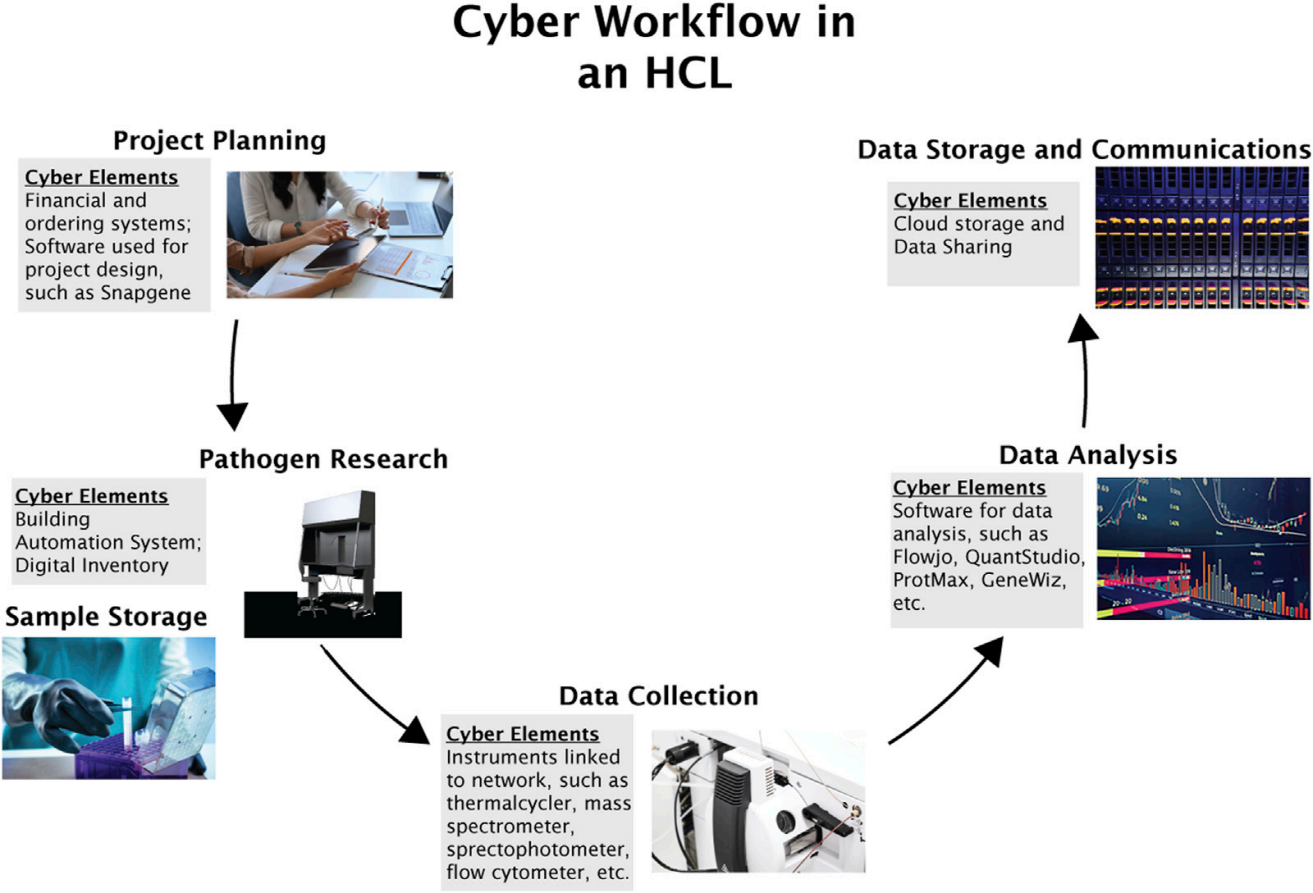
General cyber-workflow of an HCL. The figure describes six processes essential to HCL functioning: project planning, pathogen research, sample storage, data collection, data analysis, and data storage and communications.

Each step of the research process is associated with a unique set of cyber and cyberphysical elements, as shown in Figure 2.

#### Project planning

The first process in the workflow is a project planning phase. For research and biomanufacturing HCLs, this phase can include experimental design, a process which can be aided and expedited by using any number of potential software tools. For example, the software tools Snapgene and Geneious assist in the design of genetic materials for experiments (Geneious, 2022; SnapGene, 2022). In each of the types of HCLs, electronic budgets and ordering systems can assist in planning and acquiring needed materials, such as assays, personal protective equipment (PPE), genetic materials, or pathogenic samples. While simple, these systems are critical to the functioning of a laboratory. Because these systems are cyber-based, they are vulnerable to a cyber attack; furthermore, the regular downloading of various software and using online vendors may create additional entry points that malicious actors may exploit (Sarder and Haschak, 2019).

#### Pathogen research

The second process we considered is pathogen research. While some cyber and cyberphysical elements related to this step are specific to particular types of laboratories, several assets related to the handling and containment of pathogens during the research process were similar across HCL types. For example, most HCLs utilize building automation systems (BASs) to control various environmental and containment functions in addition to systems required to maintain normal operations of the laboratory. The most sophisticated BAS can control, monitor, and log data for the ventilation, pressurization parameters, temperature, containment functions, and power, all of which are important to preventing pathogen release and protecting laboratory personnel from accidental exposure (Coogan and Siemens, 2021). A BAS may also be able to monitor who enters and exits the building, ensuring the safety and security of workers by preventing unauthorized personnel from entering the facility (Siemens, 2021). These systems can have a built-in quality management function, logging data to determine the operationality of each part of the system (Siemens, 2021). While a more sophisticated BAS provides greater control over specific parameters within the laboratory and can provide increased awareness of laboratory systems by logging relevant data, the more systems in a laboratory that are connected to the BAS, the greater the attack surface and the greater the scope of potential consequences should a successful cyber attack occur.

Laboratory BASs can also control certain aspects of airflow as it pertains to biological safety cabinets (BSCs), depending on the type and class of cabinet used in the facility (Siemens, 2021). Class II/III BSCs, which are used for handling the HCPs worked with in HCLs, perform three main functions: to protect the samples from contamination, the workers from accidental exposure, and the environment from accidental contamination (MIT EHS, 2019). This is achieved through High-Efficiency Particulate Air (HEPA) filtering both intake and exhaust air and creating a negative pressure airflow under the hood of the cabinet, simultaneously preventing contaminated laboratory air from entering the workspace, preventing infectious material from flowing out of the cabinet, and preventing the exhaust of contaminated air from the BSC (WHO, 2020b). Disruptions to the airflow can occur through direct tampering with the settings on the BSC, a loss of power to the BSC, or by altering the conditions of the airflow within the laboratory or the exhaust by compromising the integrity or availability of the BAS. Even minor airflow disturbances can significantly impact the protective functions of the BSC, which are essential to preventing worker exposure, environmental contamination, and inaccurate experimental results due to sample contamination (Parks et al., 2022). While most BSCs currently in use are not connected to the internet, advances in the CPSs of laboratory equipment, including BSCs, has facilitated increased networking and internet connectivity options. Thermo Fisher recently announced the release of the Herasafe 2030i Biological Safety Cabinet, which can connect to Wi-Fi and be monitored remotely through the Thermo Fisher app (Thermo Fisher, 2021a). A BSC like this one, which is connected to the internet, is therefore also vulnerable to a direct cyber attack.

#### Sample storage

The third process we considered was sample storage and inventory management. Samples stored in HCLs naturally include HCPs. Inventory of pathogenic samples can be managed differently depending on the available resources of a laboratory, ranging from manual logs and written labels to integrated laboratory information management systems (LIMS) equipped with sample tracking software that can monitor samples and reagents throughout the workflow (Aguirre et al., 2013; Hashim and Arifin, 2013). In storage, many samples are sensitive to changes in the environment and require specific conditions to maintain the quality of the samples (Theron et al., 2003). Sample storage devices, such as freezers and incubators, must therefore maintain consistent environmental conditions such as temperature and humidity to ensure the desired growth rates and prevent contamination (Thermo Fisher, 2019). In many laboratories, sample storage devices do not connect to the internet and are managed in the laboratory. However, remote monitoring and internet-connected laboratory instruments and equipment are increasing in availability (Perkel, 2017). In the case of some storage devices, this allows personnel to set up alerts if certain environmental conditions are not within set parameters and monitor when storage is accessed, or to remotely change environmental conditions as necessary (PHC Corporation of North America, 2021). Some sample storage devices use digital security measures such as a passcode or some form of identification to access the samples and reagents, in which case the physical security of samples includes a dependence on the cybersecurity of the system (Darwin Chambers, 2022).

#### Data collection

The next process we considered was data collection, a process which is also becoming increasingly internet-connected, allowing for more sophisticated laboratory automation systems and workflows (Perkel, 2017). Depending on a given laboratory’s capabilities, certain groups of instruments can be fully automated, semi-automated, or completely nonautomated (Lippi and Da Rin, 2019). CPSs which automate data collection are increasingly common in research and diagnostic laboratories (Lippi and Da Rin, 2019). Laboratories with fully automated, cyber-connected groups of analysis instruments allow for efficient and complete analysis of samples, capable of doing several different types of tests and working with different sample types in parallel (Lippi and Da Rin, 2019). In a semi-automated laboratory, several types of tests can be run automatically, but the cyberphysical system is generally limited to one type of sample (Lippi and Da Rin, 2019). Even if workflows are not automated through sophisticated systems, individual instruments may still be cyber-connected as many instruments contain a cyber-physical element where data collection is controlled through a connected computer. Because the data collection workflow is critical to the functioning of an HCL, understanding which assets are cyber-connected and how these cyber-connected assets are networked is foundational to assessing cyber risks in an HCL.

In recent years, the rapid advancements in laboratory automation have led to unique cyberphysical systems such as a “mobile robot chemist” and other similar advances where automated robots may work with materials, chemicals, or even pathogens (Burger et al., 2020). Similar robotic aids are being used in hospitals, and it is reasonable to expect they will become more common in HCLs, especially if robots are designed to safely handle dangerous pathogens (Sashin, 2019). As these technologies are integrated into HCLs, they will bring their own cybersecurity implications because of their vulnerability to compromise due to a cyber incident.

#### Data analysis

While we distinguish data analysis and data collection as two individual processes, they are often intertwined in the laboratory as data analysis may occur directly within the programs that control instrumentation for data collection. To perform data analysis, it is common for laboratories to utilize software and third-party platforms. These programs are highly dependent on the specific type of work being performed. Still, there are countless examples of software packages for data analysis, such as Flowjo or QuantStudio, which perform analysis of flow cytometry and Polymerase Chain Reaction (PCR) experiments, respectively (FlowJo, 2022; Thermo Fisher, 2022). These tools, including an abundance of open-source tools, are cyber assets and, therefore, may be directly affected by a cyber attack.

#### Data storage and communications

The final step we considered is data storage and communications. HCLs store data relevant to significant research findings, intellectual property, or diagnostic information. For many laboratories, this stored data is of significant value to the laboratories themselves and the scientific community and can be considered the key information asset possessed by laboratories. To store this data, laboratories may utilize data storage platforms, such as GitHub or Google Drive, or their own on-premises or cloud-based data storage solution (GitHub, 2022; Google, 2023). Each of these solutions has different levels of cybersecurity and could introduce an additional attack vector through which a cyber attack could occur (Voas and Hurlburt, 2015).

As an extension of data security considerations, data sharing and communications can also introduce new vulnerabilities into the cyber-workflow of research laboratories (University of Cambridge, 2022). Research partnerships and data sharing have considerable benefits but can introduce additional vulnerabilities. Like many workplaces, communication among laboratory personnel and collaborators is often conducted via email, one of the most common attack vectors used in cyber attacks (Trend Micro, 2022). HCLs could experience a cyber incident through a compromise of one of their assets, a corrupted email sent by an unwitting colleague, or a targeted attack by a malicious actor pretending to be a colleague. Data and information sharing between partners also increases the number of devices storing valuable data, thereby increasing the attack surface and creating a potential for interception of communications.

### Cyber elements of research laboratories

Of the types of HCLs, research laboratories map most directly to the general considerations outlined above. Unique priorities within research laboratories may ascribe extra value to certain assets. For example, research data may be particularly valuable, especially if the lab possesses unique and hard-to-reproduce data sets or research findings. Compared to other types of HCLs, research data is more likely to have dual use potential, posing a greater target for a malicious actor. Research labs may also possess legacy samples and biorepositories of pathogen samples which are impossible to recreate. This inventory may be managed through cyber-connected systems. Finally, research HCLs are likely to be part of universities of other larger institutions, where these laboratories may operate within a larger institutional cyber-infrastructure. If cyber systems are connected within the broader institution, a cyber incident anywhere in the institution could impact the laboratory.

### Cyber elements of diagnostic laboratories

Diagnostic HCLs function as part of a laboratory system that requires coordination and communication between hospitals and clinics, other laboratories, and public health entities within the diagnostic network to conduct disease surveillance operations and facilitate sharing of information, samples, and resources between laboratories (Naidoo and Ihekweazu, 2020; Pabbaraju et al., 2020). The workflow of a diagnostic HCL can be summarized as receiving data and samples, storing and handling samples, collecting and analyzing sample data, and reporting results. Like research laboratories, diagnostic laboratories rely on inventory and sample storage for operations and may utilize BASs, BSCs, and third-party platforms for data management and utilize laboratory automation. While automation in research laboratories is becoming increasingly common, many diagnostic laboratories have already achieved some level of automation and therefore have more cyber-connected assets (Lippi and Da Rin, 2019). The importance of these common assets and their cybersecurity considerations are discussed in the previous section.

Cybersecurity considerations specific to the diagnostic laboratory begin when a laboratory receives a sample and accompanying metadata. Metadata can include sensitive information such as patient data [e.g., personally identifiable information (PII), protected health information (PHI)], type of sample, tests to be performed, or the location of the patient (Viswanadham, 2021). While policies and regulations differ between countries, the information obtained and used by the diagnostic laboratory is considered highly sensitive information in most countries (Bellman et al., 2004). Due to the sensitive and personal nature of the information, ensuring confidentiality is a high priority for diagnostic laboratories.

### Cyber elements of high-containment biomanufacturing facilities

A small subset of biomanufacturing facilities requires the advanced containment precautions found in HCLs to produce live-attenuated vaccines (LAVs) for pathogens such as SARS-CoV-2, *Bacillus anthracis*, and *Yersinia pestis*, the causative agents of COVID-19, anthrax, and plague, respectively (Feodorova et al., 2014; Ditchburn and Hodgkins, 2019; Goswami, 2020). A live-attenuated vaccine (LAV) is created using a live pathogen that has undergone a process reducing its ability to cause disease in a specific host (Pöyhönen et al., 2019). Thus, LAVs are created from viable pathogens and, in the case of LAVs for HCPs, may require high-containment precautions. For a review of more general cyber risks of biomanufacturing facilities, see Mantle et al. (2019) and Guttieres et al. (2019).

Like other HCLs, high-containment biomanufacturing facilities rely on inventory and sample storage for operations. They may also utilize a BAS, BSCs, third-party data platforms, and laboratory automation to increase efficiency, safety, and security within the laboratory. However, several components and unique systems within high-containment biomanufacturing facilities have special cyberbiosecurity considerations that differ from diagnostic and research laboratories.

During the upstream production process of LAVs, biomanufacturing facilities employ a number of CPSs to carry out and control processes (Arenas and Maria, 2022). Bioreactors are common CPSs used in the propagation of LAVs and are programmed with certain parameters that control conditions such as nutrient concentrations, oxygen concentrations, and dilution rate (Sha, 2021). These systems ensure proper growth rate, retention of attenuation, and prevention of contamination of the LAV stock, all of which are essential to the overall safety of the product and the safety of the workers interacting with the vaccine stock (FDA, 2017). Certain bioreactors allow for internet connection and remote monitoring, providing a potential point of entry to deliver a cyber attack (Lab Owl, 2020). Downstream processing may similarly utilize CPSs such as chromatography systems to purify the strain, removing contaminants from the vaccine stock (Arenas and Maria, 2022). Chromatographs can connect to and be monitored by networked systems, making these instruments vulnerable to cyber attacks (Thermo Fisher, 2021b).

Maintaining the integrity and availability of the production process is essential to ensure the safety and efficacy of the distributed LAV. During each step of the production process, data is routinely collected and reviewed for both quality control and research and development purposes as a part of the quality management system (QMS) (Mantle et al., 2019). Quality control management is essential to ensure the desired product is safe, free from contaminants and meets regulatory standards. Understanding the cyber-connectedness of the manufacturing and quality control systems within biomanufacturing laboratories is foundational to understanding the associated impacts.

## Identified Areas of Impact

**FIGURE 3:**
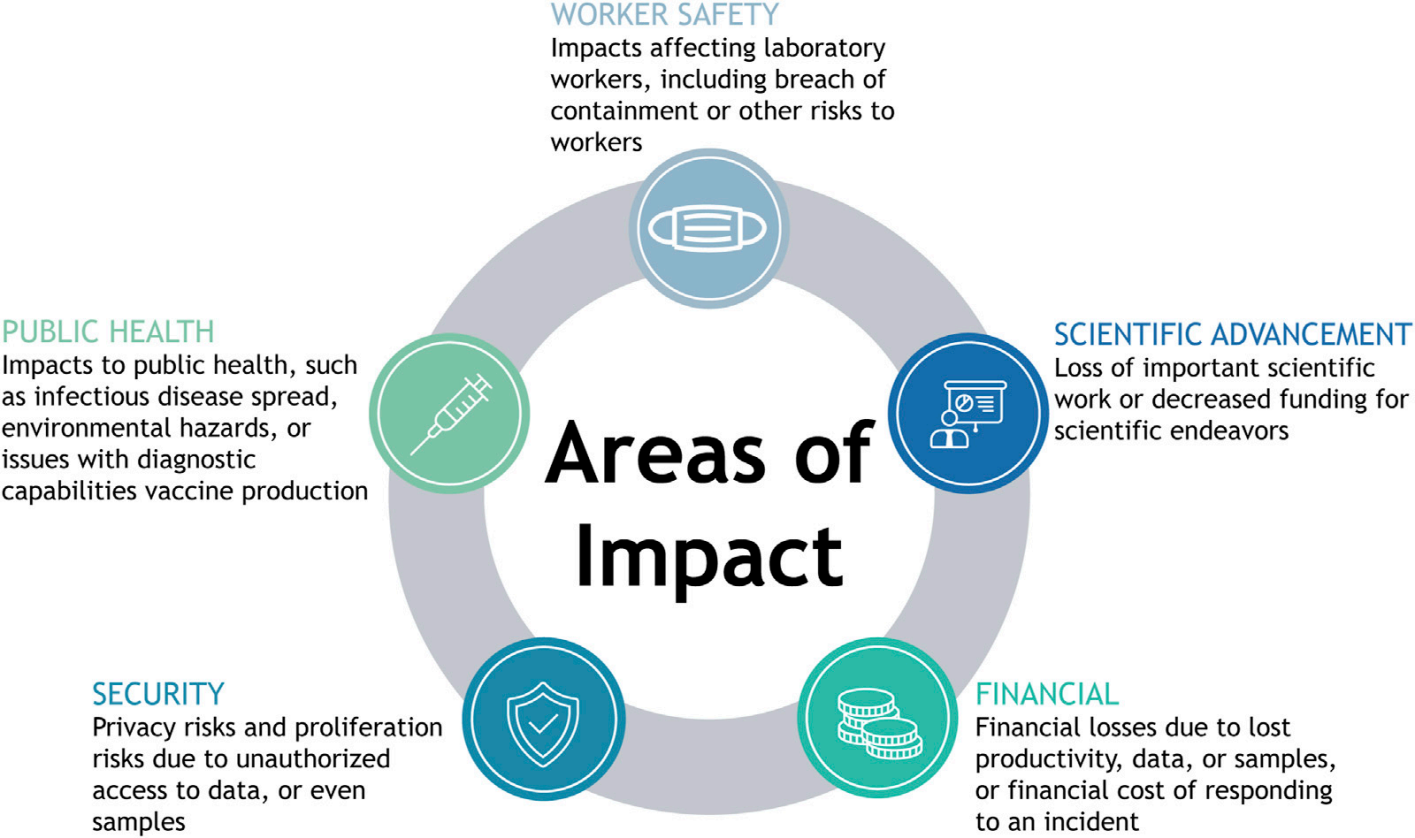
Identified areas of impact. Graphic showing areas of impact including public health, worker safety, security, scientific advancement, and financial.

The discussion above highlights the critical functions of many cyber and cyberphysical elements within HCLs. Given the critical functions of the cyber and cyberphysical systems in HCLs, a cyber incident could lead to a range of negative consequences. This section analyzes the mapped workflows in diagnostic, research, and biomanufacturing laboratories to identify the potential impacts that could occur due to a cyber incident. We first connected each asset to related potential impacts, considering losses of confidentiality, integrity, or availability of each asset due to any form of cyber attack. Upon identifying potential impacts due to the compromise of cyber and cyberphysical systems in an HCL, we found five overarching categories under which all of the identified impacts fell: worker safety impacts, public health impacts, security impacts, impacts affecting scientific advancement, and financial impacts (Figure 3). In the following section, we present the range of potential consequences due to a cyber incident in an HCL, referring to the abovementioned assets. Examples of potential forms of loss, the types of HCLs that could experience such losses, and the assets through which a cyber attack leading to each form of loss could occur are outlined in Table 1.

**TABLE 1:** Examples of Potential Forms of Loss in HCLs. The table shows selected forms of loss in HCLs within each area of impact and outlines the type(s) of HCL(s) and workflow stage(s) affected and the assets that could be compromised to result in each form of loss.

	Example loss	Lab type	Workflow stage	Asset(s)
Worker Safety	Exposure of laboratory personnel to infectious material	All	Pathogen research	BAS (containment functions), inventory management system
	Non-pathogen related worker safety risks	All	All	BAS (security and environmental functions)
Public Health	Community spread of pathogens	All	Pathogen research	BAS (containment functions)
	Loss of critical manufacturing functions	Biomanufacturing	All	Any asset that is critical to biomanufacturing facility functioning
	Misdiagnosis, or inability to diagnose	Diagnostic	Data collection, data analysis, data storage and communications	Servers/cloud-based data storage (diagnostic data), instruments, QMS
	Distribution of ineffective or unsafe materials	Biomanufacturing	Data collection, data analysis	Servers/cloud-based data storage (experimental data), instruments, QMS
	Public mistrust similar to EMA example*	Research, Biomanufacturing	Data storage and communications	Servers/cloud-based data storage, communications
Security	Unauthorized acquisition of dangerous samples from facility	All	Sample storage	BAS (security function), inventory management system, sample storage
	Unauthorized acquisition of dangerous samples during transport	All	Project planning	Financial and ordering systems
	Unauthorized acquisition of sensitive data	All	Data storage and communications	Servers/cloud-based data storage (pathogen data), communications
Scientific Advancement	Loss or corruption of large or unique datasets	Research	Data storage and communications	Servers/cloud-based data storage (large or unique datasets)
	Loss or corruption of large or unique sample sets	Research	Sample storage	BAS (security function), inventory management system, sample storage
	Public mistrust leading to loss of funding	All	All	BAS, QMS, Servers/cloud-based data storage (experimental data, diagnostic data)

*See section on previous cyber incidents in laboratories.

### Worker safety

An analysis of impacts due to the compromise of a variety of assets in an HCL revealed worker safety to be a primary area of concern in the event of a cyber incident. Worker safety considerations include consequences associated with the exposure of laboratory personnel to infectious material and consequences resulting from the physical endangerment of laboratory personnel. There are several potential attack vectors through which laboratory personnel could be exposed to infectious material. For example, a cyber incident could compromise the integrity or availability of the BAS, potentially leading to altered pressure differentials between high-hazard areas and low-hazard areas or altered airflow, which could result in the exposure of personnel to infectious material. In addition to potential exposure to infectious materials, a cyber attack on a HCL could cause other worker safety risks. For example, for laboratories with electronic locks controlled by a BAS, a cyber attack resulting in a loss of availability of the BAS when personnel are physically inside of the laboratory could result in the locking of the external electronic doors, trapping personnel inside. Another potential consequence is unauthorized access to the facility by an intentional actor or an unaware individual. This presents a physical danger to laboratory personnel and a risk to the unauthorized individual if they are unfamiliar with HCL safety procedures.

Worker safety risks may also stem from cyber incidents affecting the LIMS. An incident that compromised inventory data could leave workers unable to identify and unknowingly access dangerous samples without the appropriate protective equipment. Although no incidents of inventory corruption due to a cyber attack in an HCL are documented in the public domain, mislabeled samples have posed a risk to workers in past laboratory incidents and near-misses (Sun, 2014).

Rapid advances in robotics in the laboratory could impact worker safety. Researchers working towards integrating these evolving technologies in settings such as HCLs will need to assess the potential impacts. Depending on the role of such robots, they could also pose a risk in other categories, such as public health or scientific advancement, if a cyber incident compromised their integrity. As these advances continue, cybersecurity factors should be considered in order to protect workers who work with and around these robots.

### Public health

A successful cyber attack on an HCL also presents significant risks to public health (Table 1). Within any HCL, a cyber attack compromising the BAS-controlled ventilation and pressurization systems as described above in the worker safety section, could result in transmission within the community either through the exposure of a laboratory worker or through pathogen release. Such laboratory leaks, which can result in potential sustained pathogen transmission in the community and cause outbreaks, are prioritized in biosafety risk assessments.

In addition to the risks of laboratory-acquired infections and pathogen release, cyber attacks on diagnostic laboratories carry additional risks due to their essential role in disease surveillance and outbreak response. A cyber attack could result in the loss of availability of diagnostic capability, thereby preventing or delaying patient diagnoses. Many types of cyber incidents could disrupt workflow, including an incident compromising computer networks, a ransomware attack, an attack preventing the functioning of the BAS, or an attack that affects any of the instruments essential to the diagnostic process. Attacks that compromise essential systems may not easily be replaced or restored and could lead to significant delays in diagnosis. This could result in delays in treatment and, in the case of an outbreak, the inability to perform disease surveillance could lead to increased community transmission of disease. In addition, to delay in diagnostic capabilities, a cyber incident could affect data integrity during the diagnostic process, potentially resulting in the misdiagnosis of patients. Given the multiple cyberphysical elements in the workflow, loss of integrity could occur during data collection, data analysis, quality control, or data storage and communications. Misdiagnosis can have similar, and potentially worse, consequences compared to delays in diagnosis, including patients receiving incorrect treatments or continued transmission of diseases throughout the community. Again, these consequences can become more extreme in the event of an ongoing outbreak, when systems-wide laboratory capacity is already limited, or when a loss of data integrity goes undetected.

In addition to diagnostic laboratories, biomanufacturing facilities also perform functions essential to public health. The NotPetya cyber attack described earlier illustrates this concept (Mcquade, 2018). Briefly, Merck’s infrastructure was hit by a non-targeted cyber attack, resulting in a months-long shutdown of critical operations relating to the production of several essential drugs and vaccines (Mcquade, 2018). High-containment biomanufacturing facilities could also become a victim of such an attack, which could reduce vaccine production and slower rollout. In the case of the NotPetya attack, CDC stockpiles and other producers were able to meet demand; however, future incidents could create shortages of a vaccine or other critical medical countermeasures, resulting in increased disease spread, morbidity, and mortality (Mcquade, 2018). Furthermore, much like potential misdiagnosis in diagnostic laboratories, a cyber incident compromising the integrity of data analysis and quality control could result in delays and ineffective or unsafe vaccines. While this would most likely require a specific targeted cyber attack, the risk to public health is considerable and should be taken seriously.

Laboratory automation brings a host of risks and benefits. Automation increases the productivity, reproducibility, and throughput of a diagnostic laboratory but also introduces far more networked devices, which increases the cyber attack surface. As described above, this increases the risk of downtime and/or misdiagnosis in the laboratory and the potential issues with quality controls described above. When exploring automation solutions, laboratories should consider implementing cyber risk mitigation strategies that help maximize the benefits of these new capabilities.

Cyber attacks on HCLs could also lead to a loss in public trust, affecting public health. Many cyber attacks, whether on laboratories or other entities, are not public knowledge, shielding organizations who are victims of cyber attacks from public fallout. A publicized cyber attack on a HCL could lead to loss of public trust in that specific institution, or a loss of public trust in the public health system as a whole. Additionally, cyber attacks on biomanufacturing facilities or research laboratories involved in producing therapeutics and vaccines could lead to the deliberate release of misinformation about these interventions, as seen in the 2021 EMA attack described earlier (Cerulus, 2021). Loss of public trust could lead to decreased vaccination rates, misuse of medicines, and lower public buy-in to public health initiatives. The substantial public health benefits of HCLs highlight the importance of building fundamental cybersecurity measures into laboratory operations.

### Security risks

A common concern in pathogen research is the potential for misuse by a malicious actor, such as the generation of bioweapons. Proliferation risk may be higher for more dangerous pathogens and certain types of experiments, such as those with dual use potential. Briefly, research with dual use potential is research that is intended to benefit society but also has the potential to cause significant harm (NIH, 2014). Dual use risk may arise from materials, methods, or information. HCLs work with pathogens (materials), develop protocols to manipulate pathogens (methods), and generate data from their work (information). All of these elements may be of interest to a malicious actor seeking to misuse research and are often considered in laboratories’ biorisk management programs (Table 1).

Few potential cyber attack pathways were identified that could result in the unauthorized acquisition of dangerous samples. While unlikely, the consequences associated with a malicious actor acquiring such pathogens are high enough to warrant consideration. An actor could acquire information about pathogenic samples that a laboratory possesses and use that information to target facilities of interest to steal pathogens from storage or sample shipments. As laboratories increase their cyber sophistication, they can implement additional safeguards to securely hold sample information and improve their ability to detect illicit access to inventories.

Several cyber attack pathways were identified that could result in the unauthorized acquisition of data associated with dangerous pathogens and personal data of patients and laboratory personnel. The safeguards to prevent unauthorized access or acquisition of data are completely cyber-based. Once a cyber attack defeats the cyber safeguards and controls, there are no other mitigation measures to prevent unauthorized access or alteration of the data. Different types of data pose different risks in terms of security. Data relating to dangerous pathogen research protocols or information with dual use potential such as virulence factors, mutations that increase transmission or pathogen survival, or genetic sequences of particularly pathogenic strains, could all pose a proliferation risk if exfiltrated by a malicious actor. Many laboratory databases also contain private information of laboratory workers. Diagnostic laboratories may also hold patient-related data, including PII, PHI, genetic sequences, and test results. Securing and encrypting stored data is important for all types of HCLs, especially for diagnostic laboratories.

### Scientific advancement

Considering the critical role that HCLs play in human and zoonotic infectious disease and pathogen research, a cyber attack affecting these laboratories could significantly hamper scientific advancement. This includes loss or corruption of large or unique sets of samples or data and delays in significant research (Table 1).

Laboratories hold valuable datasets that have been compiled with significant time, expense, and effort. Many of these datasets can be analyzed with modern data science approaches to quickly identify promising therapeutic and vaccine research pathways (Aung et al., 2021). Compromise of the integrity or availability of these large or unique datasets would harm scientific advancement. For example, unauthorized alterations to the dataset could lead to significant inaccuracies in findings. Even if detected, such changes could delay scientific advancement and necessitate laborious and expensive investigations to identify and correct errors in the data. Datasets from specific time periods or datasets compiled during specific outbreaks are also unique assets that can help advance scientific discovery. These datasets are one-of-a-kind. A compromise to the integrity or availability of such a dataset, without an available backup, would be a considerable and irreplaceable loss to science.

Certain sample sets, such as large biobanks or legacy collections, incur similar unique risks to scientific advancement as those observed with large or unique datasets. The availability of a biobank could be compromised if samples are held at the wrong temperature. Cold chains and incubator controls could be impacted by a cyber attack removing power to the facility or specific rooms or compromising digitally controlled freezers and incubators. This particular consequence is exacerbated in the case of sample storage of repositories and legacy sample collections as they likely contain specific strains or certain historic samples that are irreplaceable, resulting in both a loss of general scientific knowledge and potential financial losses to the laboratory.

In addition to significant delays in research arising from a cyber attack directly, a loss of public trust could delay scientific advancement. Public trust could be affected due to a public health incident resulting from a cyber incident, a data breach, or misinformation. Loss of public trust could result in decreased funding for research or could divert funds from research leading to scientific progress to other endeavors. A similar outcome was seen following the spread of misinformation about vaccines and autism as funds were diverted from autism research to disprove the claims of the link between vaccines and autism (Pellicano and Stears, 2011). Delays in significant research, either as a result of the cyber attack or a loss of public trust, prevent scientific progress.

### Financial risks

While most of this study emphasizes the unique risks in an HCL in terms of biosafety, biosecurity, and other public health considerations, financial losses to an organization from a cyber incident provide a particularly quantitative mechanism for understanding cyberbiosecurity risk. A cyber incident is likely to result in costs associated with a loss of productivity, either due to laboratory downtime or staff time to respond to the cost. In addition to the loss of productivity, financial losses include the monetary costs incurred by an HCL in the aftermath of a successful cyber attack. Examples of financial costs of a cyber attack include legal fees, replacing lost samples or compromised equipment, or hiring Information Technology (IT) contractors. Research and biomanufacturing HCLs also could incur the loss of intellectual property, which can impact the laboratory’s competitive advantage and have financial implications. The NotPetya attack cost an estimated USD$1.4 billion, including effects from downtime, inability to produce essential vaccines, equipment and data replacement costs, and personnel response costs (Demberger, 2022).

Cyber incidents may become publicized if they cause issues such as delays in vaccine production or a loss of privacy. In many cases, organizations also have an ethical and legal responsibility to notify those whose data was compromised or those who may be otherwise impacted by the cyber incident. These incidents can damage an organization’s reputation. Academic and government research institutions generally rely on applying for grants and government funding, so a reputational loss may affect their ability to receive funding awards. While diagnostic laboratories are an essential service, a cyber incident leading to privacy issues could also cause reputational damage. A cyber incident resulting in significant publicized consequences, such as breach of containment or sample or data theft, would almost certainly lead to reputational damage, potentially affecting funding beyond the originally impacted laboratory.

Financial losses, in particular, may stem from a broad range of types of cyber attacks and a variety of different assets in the laboratory. Essentially, any cyber incident which causes a loss of productivity will result in financial loss. The severity of financial consequences is asset dependent and further depends on the value placed on each asset by the laboratory. Therefore, we did not directly relate financial losses to specific assets in Table 1 as we did in the categories above.

## Cyber risk management in HCLs

In the sections above, we identified the cyber-connected assets common to HCLs and the potential negative consequences associated with a compromise of the confidentiality, integrity, or availability, of those assets. Building upon this discussion, we turn to consider the next step in the management of cyberbiorisks: mitigation.

Risk management approaches involve first identifying and assessing risks followed by evaluating and implementing mitigation measures to reduce those risks to an acceptable risk level. The iterative processes of identification, assessment, evaluation, and mitigation of biosafety and biosecurity risks constitutes biorisk management (WHO, 2020a). Laboratories, including HCLs, use existing guidance frameworks, such as the United States CDC’s Biosafety in Microbiological and Biomedical Laboratories (BMBL) and WHO’s Laboratory Biosafety Manual (LMB), to guide the implementation of biorisk management programs at their facilities (WHO, 2020b; CDC and NIH, 2020). However, cyber and cyberphysical risks are not explicitly included in these frameworks. Increases in the adoption of network-enabled technology in HCLs create new entry points and potential pathways for malicious actors to exploit. Therefore, biorisk management programs must adapt to account for cyber and cyberphysical risks in addition to biosafety and biosecurity risks. Risk management, laboratory safety, and security experts must come together to formally define where and how cybersecurity fits into biorisk management processes in HCLs. Here, we provide a few underlying principles to guide this conversation.

In the fields of biorisk and cyber risk management, risk is generally modeled as the product of the severity of a consequence when it occurs and the likelihood of that incident occurring (Ross, 2012). The first step in integrating cybersecurity and cyber risk mitigation in HCLs is understanding that effective control implementation reduces the likelihood of an incident or the impacts of an incident if it were to occur. Ideally, a risk mitigation program reduces both likelihood and impact. The cyber risk management process for HCLs can follow a similar approach to other areas of biorisk management. Laboratory personnel should identify existing risks and implement controls to directly reduce those risks to an acceptable level (WHO, 2020b). Using a risk-based approach, risk management programs can identify explicit linkages between controls and the elements of risk—impact and likelihood. For example, consider a ransomware attack on a laboratory. Because passwords can be stolen or guessed, multi-factor authentication (MFA) makes it much less *likely* that an attacker can gain access to an information system through a compromised user account. Robust data backup and recovery systems would decrease the *impact* of a ransomware attack, allowing the laboratory to restore systems quickly with minimal downtime and cost.

This example also demonstrates the value of implementing a layered set of control systems, with well-defined benefits and tiers of implementation. Many cyber risk management frameworks include a tier of basic controls that provides common-sense protection that does not require extensive risk assessment to implement (CIS, 2021). These controls are sometimes collectively called “cyber hygiene” and are the first controls that an organization new to cybersecurity should implement as broadly as practical (NIST, 2018). Basic cyber hygiene can be considered comparable to basic laboratory safety practices that should be followed in virtually all situations (e.g., Standard Microbiological Practices). In many cases, cyber controls have been standardized so that implementation progress can be ordered, measured, and compared across organizations. One example of standardized cyber controls are the CIS Controls, which can be used to improve an entity’s cybersecurity posture in an organized fashion (CIS, 2021)^.^ The Center for Internet Security (CIS), the organization that maintains the CIS Controls, has divided all controls into three Implementation Groups (IG) (CIS, 2021). The first, known as IG1, includes the controls that an HCL starting a cybersecurity program should focus on (CIS, 2021). Other control systems have similar ways of designating the subset of those systems that fall into that category of cyber hygiene, or basic controls for early implementation (NIST, 2018).

As the cybersecurity controls that an HCL is implementing become more sophisticated, the HCL should focus on the risk-based approach described above. Similar to decision-making in other areas of biorisk management, determining appropriate controls starts with defining risk appetites and tolerances and, depending on the selected risk management approach, developing a risk register. A risk register is a list of the potential scenarios that could cause losses stated as concrete outcomes with identified categories of loss, pathways to that loss occurring, and treatment for such risks, similar to the analysis performed in this paper (Quinn et al., 2021). It is a powerful tool for an organization to reach a consensus about the risks it faces and the path to addressing them (Barrett et al., 2020). Once a risk register is created, the organization can link implementation of cybersecurity controls to the risks on the register to communicate and explain the need for the controls. Because cybersecurity controls are published and maintained as standards for which formal and auditable measurement is possible, an HCL can implement those controls and measure the implementation against recognized benchmarks. These standards could be integrated into biorisk management programs so that identified cyber risks can be connected to a given standard of control implementation against which laboratories can measure themselves. Examples may include requiring laboratories which work with high-consequence pathogens to meet a specific tier of control implementation, or to require laboratories to address specific cyber risks, such as those related to their BAS or sensitive data.

Because many aspects of cyber control implementation require organization-wide compliance, creating both awareness and buy-in from the HCL’s staff and leadership is an essential part of cyber risk management. One difficulty in creating buy-in is that when an organization effectively implements cybersecurity controls, *nothing* happens: data is *not* lost, administrative user accounts do *not* get compromised, and information systems continue to run *un*interrupted. Issues of staff buy-in stems from a lack of awareness of their personal role in the cybersecurity of the facility and a general undervaluation of risks, including biosafety, biosecurity, and cybersecurity risks, in the laboratory (Pinard and Salazar, 2010; Naseem and Conklin, 2021). Problems in leadership buy-in arise when the cost in money or convenience of implementing controls rises to a level where the organization treats cybersecurity controls purely as an unrecoverable cost center rather than measuring the value those controls return to the organization in the form of loss avoidance. For example, imposing the added inconvenience of configuring and maintaining MFA for all users may make the compromise of user accounts more difficult, but when rigorously implemented, it adds a measure of inconvenience for all the lab’s workers. Cybersecurity professionals can explain that these changes lead to greater security, but the experience of putting them in place translates to more burden in an environment where the number of account compromises was already close to zero. If an HCL has not experienced this type of compromise, the experience of adding burdens because of incidents at other laboratories or industries can lead to frustration and the conclusion that cybersecurity is not delivering value. Raising awareness of the risks associated with cyber incidents can promote responsibility among staff.

## Conclusion

This work has outlined the unique cyber elements of HCLs, identifying the cyber risks associated with these laboratories. Like most laboratories, HCLs generally have a cyber infrastructure that hosts software and data for the planning, analysis, and dissemination of their work. Many instruments for data collection are cyberphysical systems that include computers connected directly to the instruments to record and subsequently analyze data. HCLs are distinguished by the HCPs with which they work; most HCLs use CPSs such as the BAS and sometimes even cyber-connected biosafety cabinets that maintain both safety and security while handling these dangerous pathogens. Most cyber elements are shared between research, diagnostic, and biomanufacturing HCLs, but each is distinguished by the types of data, samples, and laboratory work involved; therefore, the risks associated with these cyber elements is unique for each type of facility.

Understanding the cyber elements in HCLs enables analysis of the potential cyber risks. While all organizations have the risk of financial losses from a cyber incident, HCLs are also concerned with managing risks to worker safety, public health, security, and scientific advancement. HCLs have critical functions; diagnostic and biomanufacturing laboratories are essential to meeting immediate public health needs for disease surveillance and vaccine production. Research HCLs have the potential to create long-lasting and far-reaching benefits for society. The cyber risks and impacts outlined in this paper highlight the critical importance of improving cybersecurity for these laboratories as part of public health and biosecurity efforts.

The unique intersection of cyberphysical systems and biological systems in HCLs highlights the growing importance of collaboration between biorisk management and cybersecurity practitioners. Experts from both disciplines should collaboratively identify needs and work towards building norms in the field of cyberbiosecurity. For example, future efforts could create guidance, standards, and best practices necessary to integrate cyber risk management into existing biorisk management practices.

A significant and collaborative effort is required to build awareness and cyber risk mitigation capability in laboratories. Training should help laboratory workers identify opportunities to leverage the benefits of cyber-connected infrastructure while building a practical understanding of cyber risks. Cybersecurity training could include integrating foundational concepts into existing biosafety and biosecurity training for HCL personnel and additional teaching tools and certifications specific to laboratory cybersecurity. Simultaneously, awareness-raising efforts are required to secure organizational buy-in among decision-makers, policymakers, and leaders of scientific organizations who are empowered to set policy priorities and dedicate meaningful resources to cyber risk mitigation in HCLs. Taken together, these efforts would enable HCLs to continue their impactful work in an increasingly cyber-connected environment.

## Data Availability

The original contributions presented in the study are included in the article/Supplementary Material, further inquiries can be directed to the corresponding authors.

## References

[B1] AAG Digital (2019). How often do Cyber Attacks occur? [Online]. Available at: https://aag-it.com/how-often-do-cyber-attacks-occur/.

[B2] Aguirre W. R. S. Bartolome J. P. De Torres J. E. T. Fajilan M. J. P. Mendoza E. Z. Laguador J. M. (2013). Automated laboratory item-inventory system with Barcode. Int. J. Emerg. Technol. Adv. Eng. 3 (12), 1–4.

[B3] Arenas M. Maria J. (2022). Industrial processes for vaccines production. Barcelona, Spain: Dipòsit Digital de la Universitat de Barcelona.

[B4] Aung Y. Y. Wong D. Ting D. S. (2021). The promise of artificial intelligence: A review of the opportunities and challenges of artificial intelligence in healthcare. Br. Med. Bull. 139 (1), 4–15. 10.1093/bmb/ldab016 34405854

[B5] Barrett M. Barrett M. Marron J. Pillitteri V. Y. Boyens J. Quinn S. (2020). Approaches for federal Agencies to use the cybersecurity framework. Maryland, United States: US Department of Commerce, National Institute of Standards and Technology.

[B6] Bellman S. Johnson E. J. Kobrin S. J. Lohse G. L. (2004). International differences in information privacy concerns: A global survey of consumers. Inf. Soc. 20 (5), 313–324. 10.1080/01972240490507956

[B7] Biju J. M. Gopal N. Prakash A. J. (2019). Cyber attacks and its different types. Int. Res. J. Eng. Technol. 6 (3), 4849–4852.

[B8] Brewster T. (2021). Exclusive: Hackers Break into 'biochemical systems' at Oxford university lab studying COVID-19. [Online]. Available at: https://www.forbes.com/sites/thomasbrewster/2021/02/25/exclusive-hackers-break-into-biochemical-systems-at-oxford-uni-lab-studying-covid-19/?sh=77cf49492a39.

[B9] Burger B. Maffettone P. M. Gusev V. V. Aitchison C. M. Bai Y. Wang X. (2020). A mobile robotic chemist. Nature 583 (7815), 237–241. 10.1038/s41586-020-2442-2 32641813

[B10] CDC and NIH (2020). Biosafety in microbiological and biomedical laboratories. 6th Edition.

[B11] Cerulus L. (2021). EU Medicines Agency says hackers manipulated leaked coronavirus vaccine data. [Online]. Available at: https://www.politico.eu/article/european-medicines-agency-ema-cyberattack-coronavirus-vaccine-data/.

[B12] Check Point Research (2022). Cyber security report. https://resources.checkpoint.com/cyber-security-resources/check-point-softwares-2022-security-report.

[B13] CIS (2021). Center for internet security controls version 8. Available at: https://www.cisecurity.org/controls/v8.

[B14] Coogan J. Siemens (2021). Best practices guide: Principles for building automation systems in laboratory facilities. Arlington, VA: International Institute for Sustainable Laboratories.

[B15] Darwin Chambers (2022). Laboratory incubators. [Online]. Available at: https://www.darwinchambers.com/laboratory-incubators/.

[B16] Demberger A. (2022). Merck awarded $1.4 billion for NotPetya after 5 Years of legal Battle. [Online]. Available at: https://riskandinsurance.com/merck-awarded-1-4-billion-for-notpetya-after-5-years-of-legal-battle/#:∼:text=The%20NotPetya%20attack%20destroyed%20data,resulting%20losses%20totaled%20%241.4%20billion.

[B17] Ditchburn J.-L. Hodgkins R. (2019). *Yersinia pestis*, a problem of the past and a re-emerging threat. Biosaf. Health 1 (2), 65–70. 10.1016/j.bsheal.2019.09.001

[B18] FDA (2017). Characterization and qualification of cell substrates and other biological materials used in the production of viral vaccines for infectious disease indications. Bethesda, MD: US Food and Drug Administration.

[B19] Feodorova V. A. Sayapina L. V. Corbel M. J. Motin V. L. (2014). Russian vaccines against especially dangerous bacterial pathogens. Emerg. microbes Infect. 3 (1), 1–17. 10.1038/emi.2014.82 PMC4317636 26038506

[B20] FlowJo (2022). FlowJo, home. [Online]. Available at: https://www.flowjo.com/.

[B21] Gao A. Murphy R. R. Chen W. Dagnino G. Fischer P. Gutierrez M. G. (2021). Progress in robotics for combating infectious diseases. Sci. Robotics 6 (52), eabf1462. 10.1126/scirobotics.abf1462 34043552

[B22] Geneious (2022). Geneious.com. [Online]. Available at: https://www.geneious.com/.

[B23] GitHub (2022). GitHub: Where the world builds software.

[B24] Global Research and Analysis Team, Kaspersky Lab (2014). The epic Turla operation. [Online]. Available at: https://securelist.com/the-epic-turla-operation/65545/.

[B25] Google (2023). Google personal cloud storage and file sharing platform. [Online]. Available at: https://www.google.com/drive/.

[B26] Goswami B. (2020). Covid-19 vaccines: Lets Go for it. Indian J. Med. Biochem. 24 (3), 00. 10.5005/ijmb-24-3-iv

[B27] Guttieres D. Stewart S. Wolfrum J. Springs S. L. (2019). Cyberbiosecurity in advanced manufacturing models. Front. Bioeng. Biotechnol. 7, 210. 10.3389/fbioe.2019.00210 31552236 PMC6737271

[B28] Hashim N. Arifin N. (2013). Laboratory inventory system. Int. J. Sci. Res. (IJSR) 2, 261–264.

[B29] Henriquez M. (2022). Merck wins $1.4B lawsuit over NotPetya attack. Troy, Michigan: Security Magazine.

[B30] Kessem L. (2021). Threat actors' most targeted industries in 2020: Finanace, manufacturing, and Energy. [Online]. Available at: https://securityintelligence.com/posts/threat-actors-targeted-industries-2020-finance-manufacturing-energy/.

[B31] Krüger A. Schäfers C. Busch P. Antranikian G. (2020). Digitalization in microbiology–Paving the path to sustainable circular bioeconomy. New Biotechnol. 59, 88–96. 10.1016/j.nbt.2020.06.004 32750680

[B32] Lab Owl (2020). Remote bioreactor control and lab automation capabilities have never been more critical to lab performance and safety. [Online]. Available at: https://labowl.automated-control.com/remote-bioreactor-control-and-lab-automation-capabilities-have-never-been-more-critical-to-lab-performance-and-safety/.

[B33] Lippi G. Da Rin G. (2019). Advantages and limitations of total laboratory automation: A personal overview. Clin. Chem. Laboratory Med. (CCLM) 57 (6), 802–811. 10.1515/cclm-2018-1323 30710480

[B34] Mantle J. L. Rammohan J. Romantseva E. F. Welch J. T. Kauffman L. R. McCarthy J. (2019). Cyberbiosecurity for biopharmaceutical products. Front. Bioeng. Biotechnol. 7, 116. 10.3389/fbioe.2019.00116 31214582 PMC6554447

[B35] Mcquade M. (2018). The untold story of NotPetya, the most devastating Cyberattack in history. [Online]. WIRED. Available at: https://www.wired.com/story/notpetya-cyberattack-ukraine-russia-code-crashed-the-world/(Accessed, 2022).

[B36] MDL (2017). NotPetya ransomware disrupts Merck vaccine production. [Online]. University of Hawai'i-West O'ahu. Available at: https://westoahu.hawaii.edu/cyber/regional/gce-us-news/notpetya-ransomware-disrupts-merck-vaccine-production/.

[B37] MIT EHS (2019). Biosafety cabinets. [Online]. Available at: https://ehs.mit.edu/biological-program/biological-biosafety-cabinets/#:∼:text=A%20biosafety%20cabinet%20provides%20three,contamination%20from%20unsterile%20lab%20air.

[B38] Murch R. S. So W. K. Buchholz W. G. Raman S. Peccoud J. (2018). Cyberbiosecurity: An emerging new discipline to help safeguard the bioeconomy. Front. Bioeng. Biotechnol. 39, 39. 10.3389/fbioe.2018.00039 PMC5895716 29675411

[B39] Naidoo D. Ihekweazu C. (2020). Nigeria's efforts to strengthen laboratory diagnostics-Why access to reliable and affordable diagnostics is key to building resilient laboratory systems. Afr. J. Laboratory Med. 9 (2), 1019–1025. 10.4102/ajlm.v9i2.1019 PMC7479428 32934913

[B40] Naseem S. Conklin I. (2021). Actionable cybersecurity risk management. Trends St. Cts., 69.

[B41] NIH (2014). Tools for the identification, assessment, management and responsible communication of dual use research of concern: A companion guide.

[B42] NIST (2018). Framework for improving critical infrastructure cybersecurity. Available at: https://nvlpubs.nist.gov/nistpubs/CSWP/NIST. CSWP 4162018.

[B43] Osborne C. (2021). Oxford university lab with COVID-19 research links targeted by hackers. [Online]. Available at: https://www.zdnet.com/article/oxford-university-biochemical-lab-involved-in-covid-19-research-targeted-by-hackers/.

[B44] Pabbaraju K. Wong A. A. Douesnard M. Ma R. Gill K. Dieu P. (2020). A public health laboratory response to the pandemic. J. Clin. Microbiol. 58 (8), e01110-20–e01120. 10.1128/JCM.01110-20 32513860 PMC7383562

[B45] Parks S. Hookway H. Kojima K. Bennett A. (2022). The impact of air Inflow and interfering factors on the performance of microbiological safety cabinets. Appl. Biosaf. 27 (1), 23–32. 10.1089/apb.2021.0010 36032323 PMC9402246

[B46] Pellicano E. Stears M. (2011). Bridging autism, science and society: Moving toward an ethically informed approach to autism research. Autism Res. 4 (4), 271–282. 10.1002/aur.201 21567986

[B47] Perkel J. M. (2017). The Internet of Things comes to the lab. Nature 542 (7639), 125–126. 10.1038/542125a 28150787

[B48] Peters M. A. (2012). Bio-informational capitalism. Thesis Elev. 110 (1), 98–111. 10.1177/0725513612444562

[B49] PHC Corporation of North America (2021). Laboratory incubators and growth Chambers. [Online]. Available at: http://markitbiomedical.com/knowledge-center/files/11846_2_PHCNA_Heated-Cooled_Incubator_brochure_vf.pdf.

[B50] Pinard W. Salazar C. (2010). International perspectives on mitigating laboratory biorisks. Office of Scientific and Technical Information.

[B51] Pöyhönen L. Bustamante J. Casanova J.-L. Jouanguy E. Zhang Q. (2019). Life-threatening infections due to live-attenuated vaccines: Early manifestations of inborn errors of immunity. J. Clin. Immunol. 39 (4), 376–390. 10.1007/s10875-019-00642-3 31123910 PMC7192346

[B52] Quinn S. Ivy N. Barrett M. Witte G. Gardner R. (2021). Identifying and estimating cybersecurity risk for Enterprise risk management. Gaithersburg: Natl. Inst. Stand. Technol. NIST Special Publication, 1–52.

[B53] Reed J. C. Dunaway N. (2019). Cyberbiosecurity implications for the laboratory of the future. Front. Bioeng. Biotechnol. 7, 182. 10.3389/fbioe.2019.00182 31497596 PMC6712584

[B54] Ross R. (2012). Guide for conducting risk assessments, special publication (NIST SP). Gaithersburg: National Institute of Standards and Technology.

[B55] Sarder M. Haschak M. (2019). Cyber security and its implication on material handling and logistics, 1–18. College-Industry Council on Material Handling Education.

[B56] Sashin D. (2019). Robots join workforce at the new Stanford Hospital. [Online]. Available at: https://med.stanford.edu/news/all-news/2019/11/robots-join-the-workforce-at-the-new-stanford-hospital-.html.

[B57] Sha M. (2021). Vero cell-based vaccine production: Cell lines, Media and bioreactor options. Enfield, CT: Eppendorf.

[B58] Siemens (2021). Best Practices: Building automation systems in life science and laboratory environments.

[B59] Smith Z. M. Lostri E. (2021). The hidden costs of cybercrime. San Jose, CA: McAfee.

[B60] SnapGene (2022). The future of cloning is smarter and faster. [Online]. Available at: https://www.snapgene.com/.

[B61] Sun L. H. (2014). CDC says about 75 scientists may have been exposed to anthrax. Washington DC: The Washington Post.

[B62] Thermo Fisher (2021b). Chromeleon CDS. [Online]. Available at: https://assets.thermofisher.com/TFS-Assets/CMD/brochures/BR-80076-CDS-Chromeleon-BR80076-EN.pdf.

[B63] Thermo Fisher (2022). QuantStudio real-time PCR systems. [Online]. Available at: https://www.thermofisher.com/us/en/home/life-science/pcr/real-time-pcr/real-time-pcr-instruments/quantstudio-systems.html.

[B64] Thermo Fisher (2021a). Smart and connected Herasafe and Maxisafe 2030i biological safety cabinets. [Online]. Available at: https://assets.thermofisher.com/TFS-Assets/LPD/Flyers/Connectivity-Flyer-2030iBSC.pdf.

[B65] Thermo Fisher (2019). Thermo scientific Forma environmental Chambers. [Online]. Available at: https://assets.thermofisher.com/TFS-Assets/LPD/Product-Information/BR-FORMAENVCHAMBERS-E%200919-lores%20v3.pdf.

[B66] Theron H. Venter P. Lues J. (2003). Bacterial growth on chicken eggs in various storage environments. Food Res. Int. 36 (9-10), 969–975. 10.1016/s0963-9969(03)00117-0

[B67] Trend Micro (2022). Navigating new Frontiers: Trend Micro 2021 annual cybersecurity report. [Online]. Available at: https://documents.trendmicro.com/assets/rpt/rpt-navigating-new-frontiers-trend-micro-2021-annual-cybersecurity-report.pdf.

[B68] University of Cambridge (2022). Responsible collaboration. [Online]. Available at: https://www.strategic-partnerships.admin.cam.ac.uk/managing-risks-international-engagement/responsible-collaboration.

[B69] Viswanadham N. (2021). Ecosystem model for healthcare platform. Sādhanā 46 (4), 188–213. 10.1007/s12046-021-01708-y

[B70] Voas J. Hurlburt G. (2015). Third-party Software's trust quagmire. Computer 48 (12), 80–87. 10.1109/mc.2015.372 27110033 PMC4840412

[B71] WHO (2020a). Biosafety programme management. Available at: https://apps.who.int/iris/bitstream/handle/10665/337963/9789240011434-eng.pdf.

[B72] WHO (2020b). Laboratory biosafety manual Fourth edition and associated Monographs. Geneva, Switzerland: Biosafety Program Management WHO.

[B73] Yeh K. B. Tabynov K. Parekh F. K. Mombo I. Parker K. Tabynov K. (2021). Significance of high-containment biological laboratories performing work during the COVID-19 pandemic: Biosafety level-3 and -4 labs. Front. Bioeng. Biotechnol. 9, 720315. 10.3389/fbioe.2021.720315 34485259 PMC8414973

