# Supplementary material for: Cyberbiosecurity in high-containment laboratories
Source: Front Bioeng Biotechnol. 2023 Jul 25;11:1240281. doi: 10.3389/fbioe.2023.1240281 (PMC10407794; doi:10.3389/fbioe.2023.1240281)
Supplement: Supplementary file 1 [file Table1.DOCX]

Supplemental Table 1**.** References by Section. This table includes the references used to write the historical incidents, cyber-workflow, and risk management sections of the paper.

| **Reference** | **Category** |
| --- | --- |
| Aguirre, W.R.S., Bartolome, J.P., De Torres, J.E.T., Fajilan, M.J.P., Mendoza, E.Z., and Laguador, J.M. (2013). Automated Laboratory Item-Inventory System with Barcode. *Int. J. Emerg. Technol. Adv. Eng.* 3(12)**,** 1-4. | Cyber Workflow |
| Arenas, M., Maria, J. (2022). Industrial processes for vaccines production. | Cyber Workflow |
| Bellman, S., Johnson, E.J., Kobrin, S.J., and Lohse, G.L. (2004). International differences in information privacy concerns: A global survey of consumers. *The Information Society* 20(5)**,** 313-324. | Cyber Workflow |
| Burger, B., Maffettone, P.M., Gusev, V.V., Aitchison, C.M., Bai, Y., Wang, X., et al. (2020). A mobile robotic chemist. *Nature* 583(7815)**,** 237-241. doi: 10.1038/s41586-020-2442-2. | Cyber Workflow |
| Coogan, J. and Siemens (2021). "Best Practices Guide: Principles for Building Automation Systems in Laboratory Facilities". International Institute for Sustainable Laboratories | Cyber Workflow |
| Darwin Chambers. (2022). *Laboratory Incubators* [Online]. Available: https://www.darwinchambers.com/laboratory-incubators/ [Accessed]. | Cyber Workflow |
| Ditchburn, J.-L., and Hodgkins, R. (2019). Yersinia pestis, a problem of the past and a re-emerging threat. *Biosafety and Health* 1(2)**,** 65-70. | Cyber Workflow |
| FDA. (2017). "Characterization and qualification of cell substrates and other biological materials used in the production of viral vaccines for infectious disease indications. US Food and Drug Administration, Bethesda, MD".). | Cyber Workflow |
| FlowJo (2022). *Home* [Online]. Available: https://www.flowjo.com/ [Accessed]. | Cyber Workflow |
| Feodorova, V.A., Sayapina, L.V., Corbel, M.J., and Motin, V.L. (2014). Russian vaccines against especially dangerous bacterial pathogens. *Emerging microbes & infections* 3(1)**,** 1-17. | Cyber Workflow |
| Gao, A., Murphy, R.R., Chen, W., Dagnino, G., Fischer, P., Gutierrez, M.G., et al. (2021). Progress in robotics for combating infectious diseases. *Science Robotics* 6(52)**,** eabf1462. | Cyber Workflow |
| Geneious (2022). *Geneious.com* [Online]. Available: https://www.geneious.com/ [Accessed]. | Cyber Workflow |
| GitHub (2022). Where the World Builds Software. | Cyber Workflow |
| Google *Personal Cloud Storage and File Sharing Platform* [Online]. Available: https://www.google.com/drive/ [Accessed]. | Cyber Workflow |
| Goswami, B. (2020). COvid-19 vaccines: Lets Go for it. *Indian Journal of Medical Biochemistry* 24(3)**,** 00-00. | Cyber Workflow |
| Guttieres, D., Stewart, S., Wolfrum, J., and Springs, S.L. (2019). Cyberbiosecurity in Advanced Manufacturing Models. *Frontiers in Bioengineering and Biotechnology* 7. doi: 10.3389/fbioe.2019.00210. | Cyber Workflow |
| Hashim, N., and Arifin, N. (2013). Laboratory inventory system. *International Journal of Science and Research (IJSR) Volume* 2**,** 261-264. | Cyber Workflow |
| Lab Owl. (2020). *Remote Bioreactor Control and Lab Automation Capabilities Have Never Been More Critical to Lab Performance and Safety* [Online]. Available: Lippi G, Da Rin G. (2019) Advantages and limitations of total laboratory automation: a personal overview. Clinical Chemistry and Laboratory Medicine (CCLM). 57 (6): 802-811. [Accessed]. | Cyber Workflow |
| Lippi, G., and Da Rin, G. (2019). Advantages and limitations of total laboratory automation: a personal overview. *Clinical Chemistry and Laboratory Medicine (CCLM)* 57(6)**,** 802-811. | Cyber Workflow |
| Mantle, J.L., Rammohan, J., Romantseva, E.F., Welch, J.T., Kauffman, L.R., McCarthy, J., et al. (2019). Cyberbiosecurity for Biopharmaceutical Products. *Frontiers in Bioengineering and Biotechnology* 7. doi: 10.3389/fbioe.2019.00116. | Cyber Workflow |
| MIT EHS. (2019). *Biosafety Cabinets* [Online]. Available: https://ehs.mit.edu/biological-program/biological-biosafety-cabinets/#:~:text=A%20biosafety%20cabinet%20provides%20three,contamination%20from%20unsterile%20lab%20air [Accessed]. | Cyber Workflow |
| Naidoo, D., and Ihekweazu, C. (2020). Nigeria's efforts to strengthen laboratory diagnostics-Why access to reliable and affordable diagnostics is key to building resilient laboratory systems. *African Journal of Laboratory Medicine* 9(2)**,** 1-5. | Cyber Workflow |
| Pabbaraju, K., Wong, A.A., Douesnard, M., Ma, R., Gill, K., Dieu, P., et al. (2020). A Public Health Laboratory Response to the Pandemic. *Journal of Clinical Microbiology* 58(8)**,** e01110-01120. doi: doi:10.1128/JCM.01110-20. | Cyber Workflow |
| Parks, S., Hookway, H., Kojima, K., and Bennett, A. (2022). The Impact of Air Inflow and Interfering Factors on the Performance of Microbiological Safety Cabinets. *Appl Biosaf* 27(1)**,** 23-32. doi: 10.1089/apb.2021.0010. | Cyber Workflow |
| Perkel, J.M. (2017). The Internet of Things comes to the lab. *Nature* 542(7639)**,** 125-126. | Cyber Workflow |
| PHC Corporation of North America. (2021). *Laboratory Incubators and Growth Chambers* [Online]. Available: http://markitbiomedical.com/knowledge-center/files/11846_2_PHCNA_Heated-Cooled_Incubator_brochure_vf.pdf [Accessed]. | Cyber Workflow |
| Pöyhönen, L., Bustamante, J., Casanova, J.-L., Jouanguy, E., and Zhang, Q. (2019). Life-threatening infections due to live-attenuated vaccines: early manifestations of inborn errors of immunity. *Journal of clinical immunology* 39(4)**,** 376-390. | Cyber Workflow |
| Sarder, M., and Haschak, M. (2019). Cyber security and its implication on material handling and logistics. *College-Industry Council on Material Handling Education***,** 1-18. | Cyber Workflow |
| Sashin, D. (2019). *Robots join workforce at the new Stanford Hospital* [Online]. Available: https://med.stanford.edu/news/all-news/2019/11/robots-join-the-workforce-at-the-new-stanford-hospital-.html [Accessed]. | Cyber Workflow |
| Sha, M. (2021). "Vero Cell-based Vaccine Production: Cell lines, Media and Bioreactor Options". Eppendorf. | Cyber Workflow |
| Siemens (2021). "Best Practices: Building automation systems in life science and laboratory environments". | Cyber Workflow |
| SnapGene (2022). *The Future of Cloning is Smarter and Faster* [Online]. Available: https://www.snapgene.com/ [Accessed]. | Cyber Workflow |
| ThermoFisher (2019). *Thermo Scientific Forma Environmental Chambers* [Online]. Available: https://assets.thermofisher.com/TFS-Assets/LPD/Product-Information/BR-FORMAENVCHAMBERS-E%200919-lores%20v3.pdf [Accessed]. | Cyber Workflow |
| Thermo Fisher. (2021a) *Smart and connected Herasafe and Maxisafe 2030i Biological Safety Cabinets* [Online]. Available: https://assets.thermofisher.com/TFS-Assets/LPD/Flyers/Connectivity-Flyer-2030iBSC.pdf [Accessed]. | Cyber Workflow |
| ThermoFisher (2021b). *Chromeleon CDS* [Online]. Available: https://assets.thermofisher.com/TFS-Assets/CMD/brochures/BR-80076-CDS-Chromeleon-BR80076-EN.pdf [Accessed]. | Cyber Workflow |
| ThermoFisher (2022). *QuantStudio Real-Time PCR Systems* [Online]. Available: https://www.thermofisher.com/us/en/home/life-science/pcr/real-time-pcr/real-time-pcr-instruments/quantstudio-systems.html [Accessed]. | Cyber Workflow |
| Theron, H., Venter, P., and Lues, J. (2003). Bacterial growth on chicken eggs in various storage environments. *Food Research International* 36(9-10)**,** 969-975. | Cyber Workflow |
| Trend Micro. (2022). *Navigating New Frontiers: Trend Micro 2021 Annual Cybersecurity Report* [Online]. Available: https://documents.trendmicro.com/assets/rpt/rpt-navigating-new-frontiers-trend-micro-2021-annual-cybersecurity-report.pdf [Accessed]. | Cyber Workflow |
| University of Cambridge. (2022). *Responsible Collaboration* [Online]. Available: https://www.strategic-partnerships.admin.cam.ac.uk/managing-risks-international-engagement/responsible-collaboration [Accessed]. | Cyber Workflow |
| Viswanadham, N. (2021). Ecosystem model for healthcare platform. *Sādhanā* 46(4)**,** 1-13. | Cyber Workflow |
| Voas, J., and Hurlburt, G. (2015). Third-Party Software's Trust Quagmire. *Computer* 48(12)**,** 80-87. doi: 10.1109/mc.2015.372. | Cyber Workflow |
| Biju, J.M., Gopal, N., and Prakash, A.J. (2019). Cyber attacks and its different types. *International Research Journal of Engineering and Technology* 6(3)**,** 4849-4852. | Historical Incidents |
| Brewster, T. (2021). *Exclusive: Hackers Break Into 'Biochemical Systems' At Oxford University Lab Studying COVID-19* [Online]. Available: https://www.forbes.com/sites/thomasbrewster/2021/02/25/exclusive-hackers-break-into-biochemical-systems-at-oxford-uni-lab-studying-covid-19/?sh=77cf49492a39 [Accessed]. | Historical Incidents |
| Cerulus, L. (2021). *EU Medicines Agency says hackers manipulated leaked coronavirus vaccine data* [Online]. Available: https://www.politico.eu/article/european-medicines-agency-ema-cyberattack-coronavirus-vaccine-data/ [Accessed]. | Historical Incidents |
| [Check Point Research. (2022). "Cyber Security Report". https://resources.checkpoint.com/cyber-security-resources/check-point-softwares-2022-security-report.](https://resources.checkpoint.com/cyber-security-resources/check-point-softwares-2022-security-report) | Historical Incidents |
| Demberger, A. (2022). *Merck Awarded $1.4 Billion for NotPetya After 5 Years of Legal Battle* [Online]. Available: https://riskandinsurance.com/merck-awarded-1-4-billion-for-notpetya-after-5-years-of-legal-battle/#:~:text=The%20NotPetya%20attack%20destroyed%20data,resulting%20losses%20totaled%20%241.4%20billion [Accessed]. | Historical Incidents |
| Global Research & Analysis Team, Kaspersky Lab. (2014). *The Epic Turla Operation* [Online]. Available: https://securelist.com/the-epic-turla-operation/65545/ [Accessed]. | Historical Incidents |
| Henriquez, M. (2022). "Merck wins $1.4B lawsuit over NotPetya attack", in: *Security.* | Historical Incidents |
| Kessem, L. (2021). *Threat Actors' Most Targeted Industries in 2020: Finanace, Manufacturing, and Energy* [Online]. Available: https://securityintelligence.com/posts/threat-actors-targeted-industries-2020-finance-manufacturing-energy/ [Accessed]. | Historical Incidents |
| Mcquade, M. (2018). *The Untold Story of NotPetya, The Most Devastating Cyberattack in History* [Online]. WIRED. Available: https://www.wired.com/story/notpetya-cyberattack-ukraine-russia-code-crashed-the-world/ [Accessed 2022]. | Historical Incidents |
| MDL (2017). *NotPetya Ransomware Disrupts Merck Vaccine Production* [Online]. University of Hawai'i-West O'ahu. Available: https://westoahu.hawaii.edu/cyber/regional/gce-us-news/notpetya-ransomware-disrupts-merck-vaccine-production/ [Accessed]. | Historical Incidents |
| Osborne, C. (2021). *Oxford University Lab with COVID-19 Research Links Targeted by Hackers* [Online]. Available: https://www.zdnet.com/article/oxford-university-biochemical-lab-involved-in-covid-19-research-targeted-by-hackers/ [Accessed]. | Historical Incidents |
| Smith, Z. M. and Lostri, E. (2021). "The Hidden Costs of Cybercrime". McAfee. | Historical Incidents |
| Barrett, M., Barrett, M., Marron, J., Pillitteri, V.Y., Boyens, J., Quinn, S., et al. (2020). *Approaches for Federal Agencies to Use the Cybersecurity Framework.* US Department of Commerce, National Institute of Standards and Technology. | Risk Management |
| CDC and NIH. (2020). "Biosafety in Microbiological and Biomedical Laboratories 6th Edition". | Risk Management |
| [CIS (2021). "Center for Internet Security Controls Version 8". https://www.cisecurity.org/controls/v8.](https://www.cisecurity.org/controls/v8) | Risk Management |
| Naseem, S., and Conklin, I. (2021). Actionable Cybersecurity Risk Management. *Trends St. Cts.***,** 69. | Risk Management |
| NIST (2018). Framework for improving critical infrastructure cybersecurity. *URL: https://nvlpubs. nist. gov/nistpubs/CSWP/NIST. CSWP* 4162018. | Risk Management |
| Pinard, W., and Salazar, C. (2010). "International perspectives on mitigating laboratory biorisks". Office of Scientific and Technical Information (OSTI)). | Risk Management |
| Quinn, S., Ivy, N., Barrett, M., Witte, G., and Gardner, R. (2021). Identifying and Estimating Cybersecurity Risk for Enterprise Risk Management. *Natl. Inst. Stand. Technol. NIST Special Publication***,** 1-52. | Risk Management |
| Ross, R. (2012). "Guide for conducting risk assessments, special publication (NIST SP). National Institute of Standards and Technology, Gaithersburg". | Risk Management |
| [WHO. (2020a). Biosafety programme management. https://apps.who.int/iris/bitstream/handle/10665/337963/9789240011434-eng.pdf.](https://apps.who.int/iris/bitstream/handle/10665/337963/9789240011434-eng.pdf) | Risk Management |
| WHO. (2020b). Laboratory Biosafety Manual Fourth Edition and Associated Monographs; Biosafety Program Management. *Geneva, Switzerland: WHO*. | Risk Management |
